# Supplementary figures and images for: Structure characteristics of mutation sites in two waxy alleles from Yunnan waxy maize (Zea mays L. var. certaina Kulesh) landraces
Source: PLoS One. 2023 Sep 8;18(9):e0291116. doi: 10.1371/journal.pone.0291116 (PMC10490952; doi:10.1371/journal.pone.0291116)

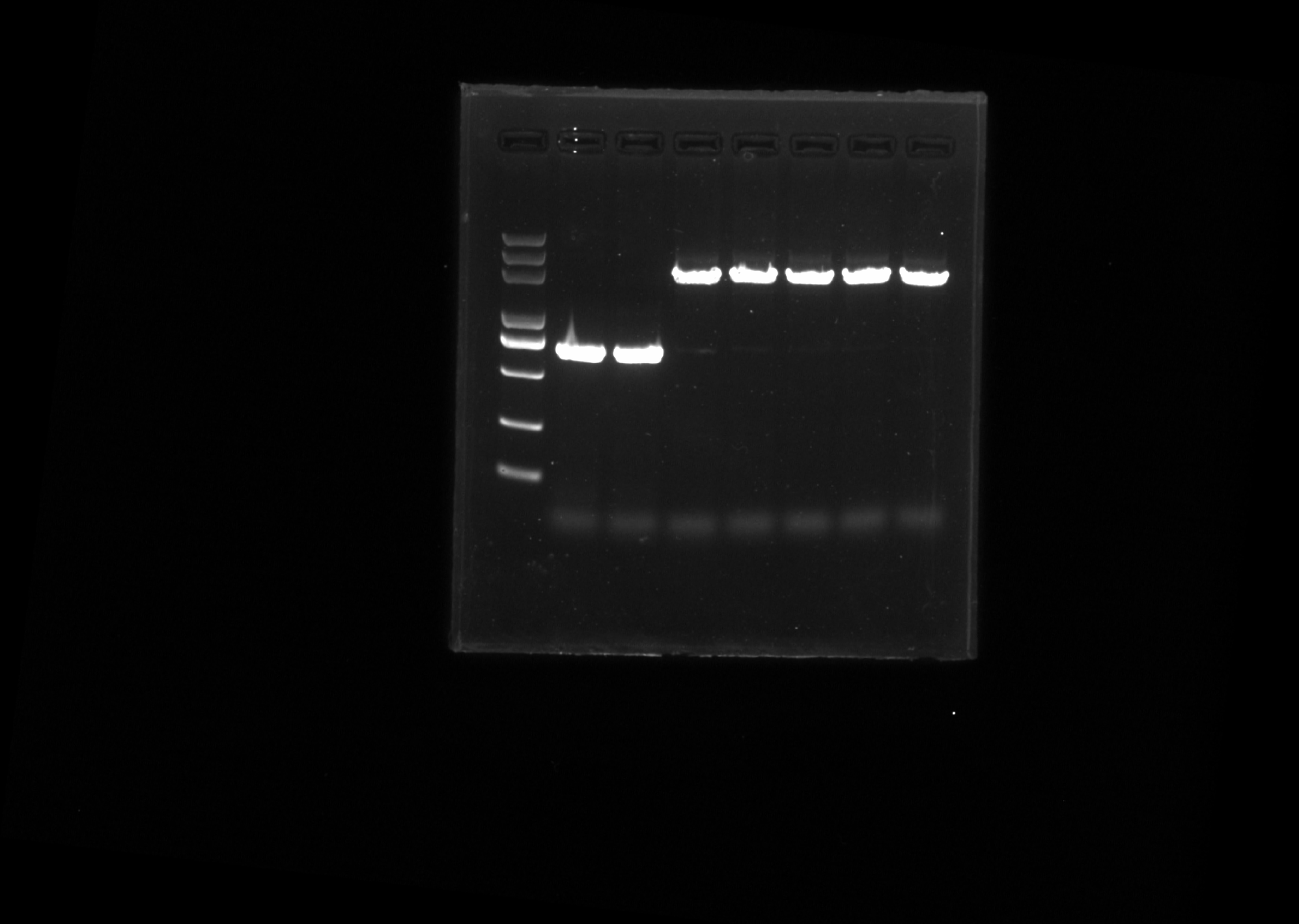

Supplement: S1 Raw images — (ZIP) [file pone.0291116.s001.zip › Fig 7A_raw_images.tif]

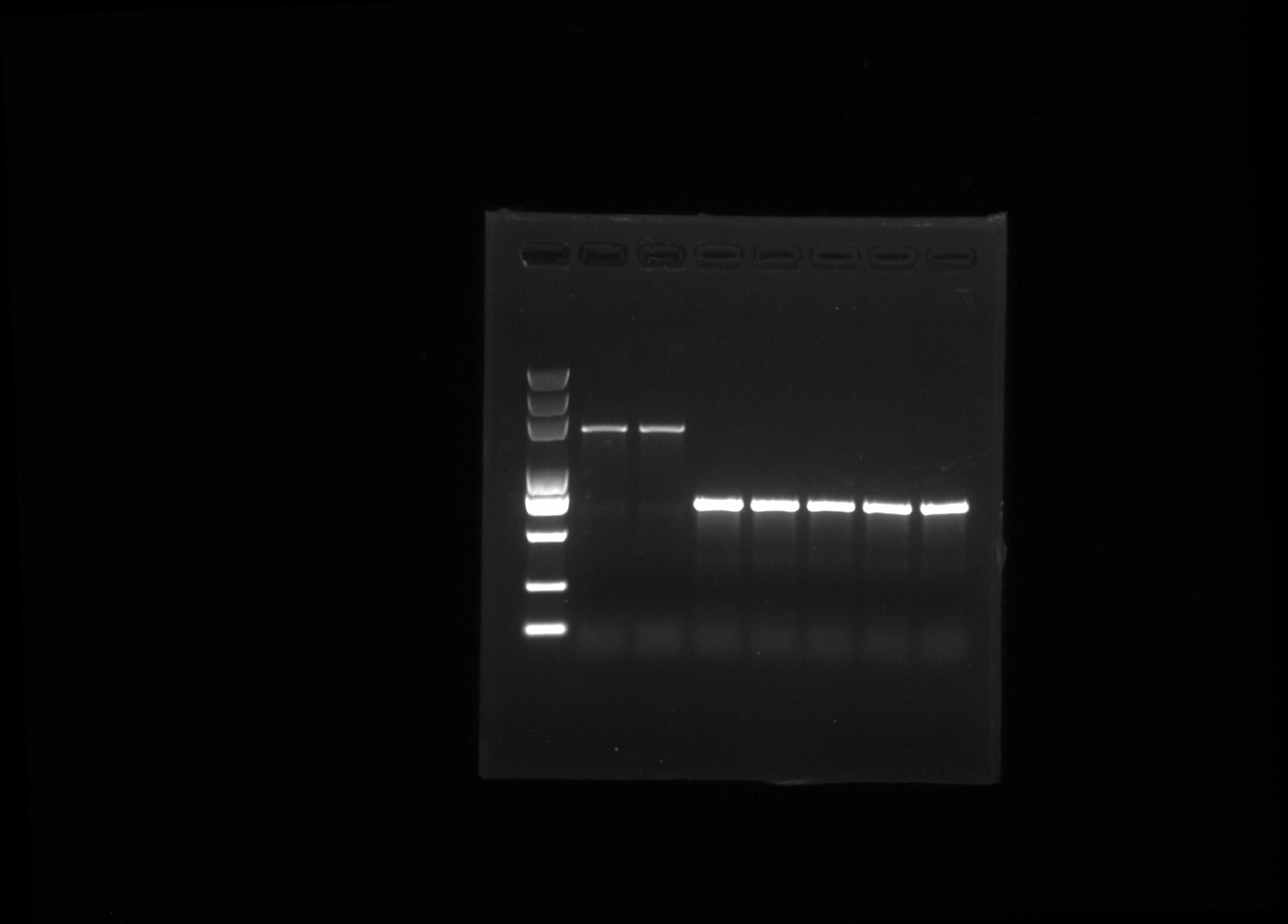

Supplement: S1 Raw images — (ZIP) [file pone.0291116.s001.zip › Fig 7B_raw_images.tif]
