# Supplementary material for: Structure characteristics of mutation sites in two waxy alleles from Yunnan waxy maize (Zea mays L. var. certaina Kulesh) landraces
Source: PLoS One. 2023 Sep 8;18(9):e0291116. doi: 10.1371/journal.pone.0291116 (PMC10490952; doi:10.1371/journal.pone.0291116)
Supplement: Attachment — Submitted filename: manuscript 20230329-R.docx [file pone.0291116.s002.docx]

**Structure Characteristics of** **Mutation Sites in Two *Waxy*** **Alleles from Yunnan Waxy** Maize (*Zea mays* L.) **Landraces**

Tingting Sun ^1,2^, and Xiaoyang Wu ^2,*^

1	State key laboratory of rice biology and breeding, China National Rice Research Institute, Chinese Academy of Agricultural Sciences, Hangzhou 310006, China.

2	Biotechnology and Germplasm Resources Institute, Yunnan Academy of Agricultural Sciences, Kunming 650223, China.

*	Correspondence: xtwxy1@163.com

**Abstract**

A large number of waxy maize landraces are distributed in Yunnan and surrounding areas, and abundant *waxy* alleles of different types are distributed in these landraces.

The identification of *waxy* alleles is helpful to the protection and utilization of these waxy landraces. This study introduced structure characteristics of *waxy* genes from two specific landraces of Yunnan, Zinuoyumi and Myanmar Four-Row Wax. Zinuoyumi has two *waxy* alleles *wx-Cin4* and *wx-Cin4-2*; Myanmar Four-Row Wax has three *waxy* alleles *wx-D10*, *wx-Reina* and *wx-D11*. The *wx-Cin4-2* and *wx-D11* are two types of *waxy* alleles first reported in this study. The *wx-Cin4-2* has two mutation sites, deletion of 30 bp in exon 10, insertion of a 1,267 bp non-long terminal repeat (non-LTR) retrotransposon *Cin4* in intron 10, and 13 bp extra sequence were found at 5’ end of the *Cin4*; the mutation site of *wx-D11* is a 1,082 bp deletion from exons 11 to 14 of *the waxy* gene and is replaced with a 72 bp filler sequence. This study enriched the type of *waxy* allele from Yunnan waxy maize landraces and further discussed the molecular basis for the formation of mutation sites of *wx-Cin4-2* and *wx-D11*.

**Keywords:** *Cin4*; Extra sequence; Filler sequence, Maize (*Zea mays* L.); Waxy maize landraces; *Waxy*, Yunnan; allele;

**Introduction**

Maize (*Zea mays* L.) originated in South America and was introduced to China ~400-500 years ago [1]. In Southwest China, residents have the habit of waxy food. Maize is under selection pressure since the introduction, and then waxy maize (*Zea mays* L. var. *certaina* Kulesh) forms a branch of maize [2]. Amylopectin is almost all starch in the endosperm of waxy maize, which makes the grain have a waxy taste [3, 4]. The waxy taste is controlled by a recessive *waxy* gene. The maize *waxy* gene is located on chromosome 9, with a total length of 4.5-Kb and contains 14 exons [5].

Yunnan is the province with the most ethnic groups in China, which is located on the Yunnan-Guizhou Plateau, bordering Myanmar, Laos and Vietnam. There is a view that Yunnan and its surrounding areas are the origin center and genetic diversity center of Chinese waxy maize [6-9]. There are abundant waxy maize landraces [10], such as an ancient landrace termed Four-row Wax due to only four rows of seed set in the cob was collected from Menghai County of Yunnan in 1970, which has been planted by the local Dai minority since 1890 and is a primitive cultivar with many characters similar to that of wild species [11, 12].

And abundant *waxy* alleles are distributed in these landraces of Yunnan *(*Figure 1). The known alleles include *wx-D7*, *wx-D10*, *wx-Cin4*, *wx-124*, *wx-Reina*, *wx-Xuanwei*, *wx-PIF/Harbinger*, *wx-hAT*, and *wx-Elote2* [13-16]; *wx-D7* and *wx-D10* were mutations caused by sequence deletion in *waxy* gene [17, 18]; *wx-Cin4*, *wx-124*, *wx-Reina*, *wx-Xuanwei*, *wx-PIF/Harbinger*, *wx-hAT*, and *wx-Elote2* are mutations caused by transposon insertion in *the waxy* gene [13-16]; *wx-D10* and *wx-Reina* are widely distributed *waxy* alleles in waxy maize landraces of Yunnan area [16, 19]. The *wx-D10* mutation site is a 30 bp deletion in the tenth exon of *the waxy* gene [17, 18]. The *wx-Reina* mutation site is the insertion of a 5.4 Kb long terminal repeat (LTR) retrotransposon *Reina*, into the tenth intron of the *waxy* gene [14].

The identification of *waxy* alleles is helpful to the protection and utilization of these waxy landraces. So far, a large number of Yunnan waxy maize landraces still contain unknown *waxy* alleles that need to be identified. In this study, we introduced structure characteristics of *the waxy* gene from two specific landraces of Yunnan, Zinuoyumi and Myanmar Four-Row Wax.

**Materials and methods**

**Plant material**

In this study, 405 waxy maize landraces from different regions of Yunnan were used as research material mentioned in our previous work [16]. The maize inbred line B73 was used as the non-waxy maize control. Seeds of research material were sampled from the Yunnan Provincial Crops Genebank of Yunnan Academy of Agricultural Sciences, P.R.China, and planted at Songming experimental field. I_2_/KI staining was used to identify the waxy grain of maize [20].

**Identification of mutation sites of *waxy* gene**

Genomic DNA was extracted from young leaves and used for *waxy* gene detection [21]. Specific markers are used to identify known *waxy* alleles (Table 1); Molecular markers that can cover the entire *waxy* gene are used to identify unknown *waxy* alleles (Table 2). PCR products were used for 1% agarose gel electrophoresis and direct sequencing.

**Sequence analysis**

Gene structure was drawn using Gene Structure Display Server (GSDS) software http://gsds1.cbi.pku.edu.cn/ [22, 23]. BlastN in NCBI is used to analyze the type of insertion sequences and find reference sequences, and CDD is used to analyze the domain of transposons [24]. ORFfinder in NCBI is used to analyze the open reading frame (ORF). Soft-ware Clustal X (1.8) was used for sequence alignment [25]. The genome sequence of the maize inbred line, B73 (http://ftp.maizesequence. org/current/assembly/) [26], was used to identify copy number of transposon with blast tool (version 2.2.23+) [27]. The secondary structure of the *waxy* gene was predicted using RNA structure 6.2 software [28]; the neighbor-joining (NG) tree was constructed using Mega 5 software [29]. The primer design software was Primer3 [30].

**Results**

This study identified the *waxy* allele composition of the Yunnan-specific waxy maize landraces Zinuoyumi and Four-Row Wax.

**Zinuoyumi**

Zinuoyumi from Lancang County (Figure 2), Yunnan Province, except purple seed and cob, the purple pigment was deposited in the whole plant during the adult stage. It is an ideal purple gene source in black waxy maize breeding (Figure 3). It is the typical waxy maize with purple seed and cob among all landraces of purple waxy maize in Yunnan, so it is selected as one of the specific landraces used in this study.

Zinuoyumi has two *waxy* alleles, *wx-Cin4* and *wx-Cin4-2*. Unlike the known *waxy* alleles, *wx-Cin4-2* has two different mutation sites, 30bp deletion in exon 10 same as *wx-D10* and 1,267 bp insertion of *Cin4* transposon in intron 10 (Figure 1). However, the allele *wx-D10* was not found in this material. It is the first time that *waxy* alleles with two mutation sites have been identified in waxy maize landraces of the Yunnan area.

*Cin4* is a class of non-long terminal repeat (non-LTR) retrotransposon in the maize genome [31]. The full-length *Cin4* transposon is about 7Kb in length and has two open reading frames (ORF) to encode the necessary enzymes for jumping in the maize genome. Non-full-length *Cin4* is usually truncated at the 5’- end of the full-length *Cin4*. Both full-length and non-full-length *Cin4* have target site duplications (TSDs) sequence and poly (A) tail at 3’- end. The length of *Cin4* in *wx-Cin4-2* is 1,267 bp, which is a truncated transposon of *Cin4*(Figure 4); The 5’- end contains part of the sequence of the second ORF of the full-length *Cin4*, and its 3’ - end is a poly (A) tail of 9 bp; Its TSDs sequence is 5’-GCAACGCGATGGATAA-3’.

In total 53 *Cin4* insertion sites were identified from the B73 genome, of which 23 had complete TSDs structure (Table 3). Subsequently, the phylogenetic tree was established with 23 *Cin4* sequences, and The *Cin4* of *wx-Cin4*, *wx-Cin4-2* and *Cin4-2* were clustered into a single clade (Figure 5). The *Cin4-2* is a full-length *Cin4* with 7,199 bp, which has the complete structure of *Cin4* and is used as the reference sequence in this study. Furthermore, the sequences of *Cin4* in *wx-Cin4* and *wx-Cin4-2* were compared with *Cin4-2*, and the 5' end sequences of both *Cin4* in *wx-Cin4* and *wx-Cin4-2* were found not to match *Cin4-2* completely, which are extra sequences of the *Cin4* (Figure 6). We found that the extra sequences come from the *Cin4* sequence itself, and there are overlapping sequences at the junction of the extra sequences with the target site sequence and with the *Cin4* sequence. The extra sequence of *Cin4* in *wx-Cin4-2* is 5'-ATAAGGGAGGTCT-3', the overlapping sequence at the junction with *wx-Cin4-2* is 5'-T-3’, and the overlapping sequence with TSD is 5'-ATAA-3' (Figure 6).

The *wx-Cin4-2* has been identified only in Zinuoyumi. Two *waxy* alleles caused by *Cin4* transposon insertion were identified in Zinuoyumi, suggesting that maybe active *Cin4* transposons exist in the genome of Zinuoyumi.

**Myanmar** **Four-Row Wax**

Four-Row Wax has long been regarded as one of the specific waxy maize landraces in the Yunnan area because its rows number of ears is four (Figure 3). Four-Row Wax was first found in the Dai nationality residential area of Menghai, Yunnan Province, and it is also distributed in other areas (Figure 2). Yunnan provincial repository for corps germplasm now stores Four-Row Wax collected from the Menghai, Menglian, Yingjiang and Namhkan areas of the China-Myanmar border, and the Four-Row Wax from Namhkan was named Myanmar Four-Row Wax by us.

It was previously reported that the *waxy* gene of Four-Row Wax is *wx-D10* [17, 18, 32]. However, our study found that Myanmar Four-Row Wax has three different alleles, including *wx-D10*, *wx-Reina* and *wx-D11* which is a new mutation found in this study. Through sequence alignment with the wild-type *waxy* gene, it was found that the *wx-D11* is a 1,082 bp deletion mutation from exon 11 to exon 14 (Figure 1), and replaced by a 72 bp filler sequence. The filler sequence has an 11 bp TSDs structure like to transposon of the genome, and the sequence is 5’-TGGTACGTGTG-3’. The filler sequence between TSDs can be divided into three parts: Part I is an 18 bp forward sequence from exon 10 of *waxy* gene; Part II is a 17 bp unknown source sequence; Part III is a 37 bp reverse complementary sequence from exon 14 of *waxy* gene (Figure 7). In addition, we noted that the mutation site of *wx-D11* was consistent with *wx-Reina*, but the TSDs sequence was not the same as *wx-Reina*.

Specific markers for *wx-Cin4-2* and *wx-D11* were further developed in this study, and agarose gel showed amplicons from Zinuoyumi and Myanmar Four-Row Wax (Figure 8).

**Discussion**

**Yunnan and its surrounding areas are the secondary origin center of waxy maize**

Vavilov’s theory on the origin center of crops: The primary origin center is the primary origin center. When the crops spread to a certain range, they will form a new recessive gene-controlled diversification area, namely the secondary origin center or secondary gene center, due to the self-crossing and natural isolation of the crops. The secondary origin also has four characteristics: (1) no wild ancestors; (2) there are new specific types; (3) there is a lot of variation; (4) there are a lot of recessive genes.

So far, it can be seen that there is no wild ancestor of maize in the Yunnan region; however, a large number of waxy maize resources were accumulated in this area with recessive waxy genes. Some of them are unique to this region, and these materials have special morphological characteristics; abundant recessive gene variation was observed from the *waxy* site. In addition, we suggest that the phenomenon of multiple alleles is a major molecular characteristic of ancient landraces, and the accumulation of *waxy* alleles is caused by selection pressure in Yunnan waxy landraces.

**The influence of Chinese waxy food culture on crop selection**

Maize originated in the Americas continent and was introduced to China about 400-500 years ago [1, 33]. It is generally believed in the academic community that waxy maize is formed by selection after gene mutations in common maize [34]. Waxy maize has been planted in China for over 200 years [6]. Maize undergoes genetic mutations within one to two hundred years after being introduced into China. These mutations were noticed by Zhuang, Dong, Buyi, Dai and other ethnic minorities in southwest China who lived in the "waxy rice cultivation area" or "waxy rice cultural circle" or "waxy food cultural circle", and these mutations were selected and retained. These ethnic minorities were developed from their ancestors, the Baiyue people, during the Ming and Qing dynasties. The Baiyue people were the earliest rice cultivators in China, as well as the earliest waxy rice cultivators and eaters. These ethnic minorities have learned and borrowed from their ancestors' experience and knowledge in the selection of waxy rice for the selection of waxy maize [35, 36]. The waxy mutant of maize, waxy maize, can be preserved and accumulated, forming hundreds of landraces or germplasm resources. Therefore, the waxy food culture and knowledge of ethnic minorities in southwest China, especially in Yunnan and surrounding areas, is the main driving force for the formation of waxy maize in China.

**Transposon activity is the main driving force for the formation of *waxy* allele diversity**

Transposon insertion is a major form of *waxy* gene mutation in maize. Transposons can be divided into DNA transposons and RNA transposons based on the difference in the transposon jumping medium within the genome [37]. RNA transposons can further divide into long terminal repeat (LTR) retrotransposon and non-LTR retrotransposon. Compared with non-LTR retrotransposon, LTR retrotransposon has long forward repeat LTR at 5'-end and 3'-end. Non-LTR retrotransposons usually have a 3'-poly (A). Whereas LTR retrotransposon can be further divided into Ty1-*Copia* and Ty3-*Gypsy* according to the order of their domains [38]. Miniature inverted-repeat transposable element (MITE) is a kind of DNA transposon which is mainly non-self-transposable [39, 40]. The insertion of different types of transposons represents the structural characteristics of different types of *waxy* alleles. The *waxy* gene mutation type of waxy maize in Yunnan province involves the above-mentioned transposons (Table 4). The mutations of *wx-Cin4-2* were also caused by transposon activity. This further indicates that the activity of transposon is the main cause of the formation of *the waxy* allele.

**Formation of an extra sequence of *Cin4* and its biological role**

Insertion of the non-LTR retrotransposon into chromosomal DNA is reported to be initiated by a mechanism called target-primed reverse transcription (TPRT), in which the target DNA was cleaved to generate a free hydroxyl (OH). This hydroxyl acts as a primer for reverse transcription using retrotransposon RNA as a template [41, 42]. However, more details of the second strand synthesis are not very clear [43]. In some reports, the following step of transposition was the cleavage of the second strand. The newly synthesized cDNA jumps from the retrotransposon RNA to the second strand at the target site. The second-strand cleavage creates a primer for second-strand synthesis. The primer was a microhomology (MH) sequence between the 5’-end of non-LTR retrotransposon and its target site [42, 44]. The formation of an extra sequence can change the terminal sequence of transposons, which is conducive to the formation of MH sequences and promotes template switching (Figure 9).

**Formation of *wx-D11* mutation site.**

Previous studies have found that *wx-B*, *wx-BI*, *wx-B6*, and *wx-C4* are mutations due to *waxy* gene sequence deletions from exons 1 to 7, and the deletion sequences are replaced by filling sequences [45]. These structures are the result of more than one molecular mechanism in the genome. One possibility is that the sequence of the deletion site can form a stable secondary structure. The secondary structure cannot be effectively opened during DNA replication. During the DNA replication process, the absence of replication results in the deletion of the DNA sequence at this site. The deletion of sequences triggers the repair mechanism of the genome, and the repair process introduces filling sequences [45]. We analyzed the secondary structure of the deletion sequence of *wx-D11* and found that this site could form a typical secondary structure (Figure 10). The other is that, DNA transposition can carry some of its flanking sequences jump to other parts of the genome; sometimes the transposition could induce double-strand DNA breakage, and then the homologous recombination repair mechanism of chromosome would be triggered to repair the DNA in this position, the repair may not be so perfect, caused the formation of filler sequence at the break sites [46].

**Author Contributions**

All authors performed experiments and wrote the manuscript. All authors have read and agreed to the published version of the manuscript.

**Funding**

This work was supported by the National Natural Science Foundation of China (32060459).

**Competing Interests**

The authors declare no competing interests.

**Reference**

1. Cao L (2004) A Summary of the Research on the Introduction of American Cereal Crops into China in Ming and Qing Dynasties. Ancient and Modern Agriculture 2:95-103 (In Chinese)
2. Liu ZZ, Song YC, Shi YS, Cai YL, Cheng WD, Qin LQ, Li Y, Wang TY (2010) Racial Classification and Characterization of Maize Landraces in China. Scientia Agricultura Sinica 43(5):899-910 (In Chinese)
3. Weatherwax P (1922) A rare carbohydrate in waxy maize. Genetics 7: 568
4. Demerec M (1924) A case of pollen dimorphism in Maize. American J Bot 11: 461-464
5. Klösgen R, Gierl A, Schwarz-Sommer Z, Saedler H (1986) Molecular analysis of the *waxy* locus of *Zea mays*. Mol Gen Genet 203: 237-244
6. Zeng MQ (1987) Blood relationship of Chinese waxy maize. China Seeds 3:21-24 (In Chinese)
7. Zeng MQ, Yang TX, Wang P (1981) The relative analyses of maize cultivar Menghai four-row wax. Acta Genet Sin 8:91-96 (In Chinese)
8. Yang TX, Zeng MQ, Wang P (1981) Analysis on peroxidase isozymes of waxy maize from South China. Acta Bot Sin 23:110-115 (In Chinese)
9. Tian ML, Huang YB, Liu YJ, Rong TZ (2003) Genetic difference between *Zea mays* sinensis and *Zea mays* indurata from Guizhou and Yunnan province revealed by SSR markers. Journal of Sichuan Agricultural University 21:213-216 (In Chinese)
10. Lei YT, Long WJ, Zhou GY, Cai Q, Wu SY (2016) Research and utilization of waxy maize germplasm resources in Yunnan. Journal of Henan Agricultural Sciences 45:1-7 (In Chinese)
11. Zeng MQ, Yang TX, Wang P (1981) The relative analyses of maize cultivar Menghai four-row wax. Acta Genet Sin 8: 91–96. (In Chinese).
12. Zhang JY, Zhang JH, Yang XH (2007) Study of genetic variations in the Xishaungbanna waxy corn landraces by SSR Analysis. Scientia Agric Sin 40: 234–243. (In Chinese).
13. Wu XY, Chen D, Lu YQ, Liu WH, Yang XM, Li XQ, Du J, Li LH (2017) Molecular characteristics of two new *waxy* mutations in China waxy maize. Mol Breeding 37: 27
14. Wu XY, Wu SY, Long WJ, Chen D, Zhou GY, Du J, Cai Q, Huang XQ (2019) New *Waxy* Allele *wx-Reina* Found in Chinese Waxy Maize. Genetic Resources and Crop Evolution 66: 885-895
15. Wu XY, Long WJ, Chen D, Zhou GY, Du J, Wu SY, Cai Q (2020) Molecular Characteristics of *Waxy* Allele *wx-xuanwei* in Yunnan Waxy Maize Landraces. Acta Agriculturae Jiangxi 32: 35-41 (In Chinese)
16. Wu XY, Long WJ, Chen D, Zhou GY, Du J, Wu SY, Cai Q (2022) *Waxy* allele diversity in waxy maize landraces of Yunnan Province, China. Journal of Integrative Agriculture. 21(2): 2-9
17. Fan LJ, Quan LY, Leng XD, Guo XY, Hu WM, Ruan SL, Ma HS, Zeng MQ (2008) Molecular evidence for post-domestication selection in the *Waxy* gene of Chinese waxy maize. Mol breeding 22:329-338
18. Fan LJ, Bao JD, Wang Y, Yao JQ, Gui YJ, Hu WM, Zhu JQ, Zeng MQ, Li Y, Xu YB (2009) Post-domestication selection in the maize starch pathway. PloS One 4: e7612
19. Yao JQ, Bao JD, Zhu JQ, Gui YJ, Shen QF, Hu WM, Fan LJ (2013) Genetic diversity of *Waxy* gene in Chinese glutinous maize. Acta Agronomica Sinica 39:43-49 (In Chinese)
20. Liu J, Rong TZ, Li WC (2007) Mutation loci and intragenic selection marker of the granule-bound starch synthase gene in waxy maize. Mol Breed 20: 93-102
21. Doyle JJ, Doyle JL (1990) Isolation of plant DNA from fresh tissue. Focus 12:13-15
22. Guo AY, Zhu QH, Chen X, Luo JC (2007) GSDS: a gene structure display server. Hereditas 29:1023–1026 (In Chinese)
23. Hu B, Jin JP, Guo AY, Zhang H, Luo JC, Gao G (2015) GSDS 2.0: an upgraded gene feature visualization server. Bioinformatics 31(8): 1296-1297
24. Marchler-Bauer A, Bo Y, Han LY, He JN, J. Lanczycki C, Lu SN, Chitsaz F, K. Derbyshire M, C. Geer R, R. Gonzales N, Gwadz M, I. Hurwitz D, Lu F, H. Marchler G, S. Song J, Thanki N, Wang ZX, A. Yamashita R, Zhang DC, Zheng CJ, Y. Geer L, H. Bryant S (2017) CDD/SPARCLE: functional classification of proteins via subfamily domain architectures. Nucleic Acids Res 45:D200-D203
25. Thompson J, Higgins D, Gibson T (1994) CLUSTALW: improving the sensitivity of progressive multiple sequence alignment through sequence weighting, position-specific gap penalties and weight matrix choice. Nucleic Acids Res 22:4673
26. S. Schnable P, Ware D, S. Fulton R, C. Stein J, Wei FS, Pasternak S, Liang CZ, Zhang JW, Fulton L, A. Graves T, Minx P, Reily AD, Courtney L, S. Kruchowski S, Tomlinson C, Strong C, Delehaunty K, Fronick C, Courtney B, M. Rock S, Belter E, Du FY, Kim K, M. Abbott R, Cotton M, Levy A, Marchetto P, Ochoa K, M. Jackson S, Gillam B, Chen WZ, Yan L, Higginbotham J, Cardenas M, Waligorski J, Applebaum E, Phelps L, Falcone J, Kanchi K, Thane T, Scimone A, Thane N, Henke J, Wang T, Ruppert J, Shah N, Rotter K, Hodges J, Ingenthron E, Cordes M, Kohlberg S, Sgro J, Delgado B, Mead K, Chinwalla A, Leonard S, Crouse K, Collura K, Kudrna D, Currie J, He RF, Angelova A, Rajasekar S, Mueller T, Lomeli R, Scara G, Ko A, Delaney K, Wissotski M, Lopez G, Campos D, Braidotti M, Ashley E, Golser W, Kim H, Lee S, Lin JK, Dujmic Z, Kim W, Talag J, Zuccolo A, Fan CZ, Sebastian A, Kramer M, Spiegel L, Nascimento L, Zutavern T, Miller B, Ambroise C, Muller S, Spooner W, Narechania A, Ren LY, Wei SR, Kumari S, Faga B, J. Levy M, McMahan L, Buren PV, W. Vaughn M, Ying K, Ye CT, J. Emrich S, Jia Y, Kalyanaraman A, Hsia AP, W. Brad B, S. Baucom R, P. Brutnell T, C. Carpita N, Chaparro C, Chia JM, Deragon JM, C. Estill J, Fu Y, A. Jeddeloh J, Han YJ, Lee H, Li PH, R. Lisch D, Liu SZ, Liu ZJ, Nagel DH, C. McCann M, SanMiguel P, M. Myers A, Nettleton D, Nguyen J, W. Penning B, Ponnala L, L. Schneider K, C. Schwartz D, Sharma A, Soderlund C, M. Springer N, Sun Q, Wang H, Waterman M, Westerman R, K. Wolfgruber T, Yang LX, Yu YS, Zhang LF, Zhou SG, Zhu QH, L. Bennetzen J, R. Kelly D, Jiang JM, Jiang N, G. Presting G, R. Wessler S, Aluru S, A. Martienssen R, W. Clifton S, W. Richard M, A. Wing R, K. Wilson R (2009) The B73 maize genome: complexity, diversity, and dynamics. Science 326:1112-1115
27. Stephen FA, Thomas LM, Alejandro AS, Zhang JH, Zhang Z, Webb M, David JL (1997) Gapped BLAST and PSI-BLAST: a new generation of protein database search programs. Nucleic Acids Res. 25:3389-3402
28. Reuter, Mathews (2010) RNAstructure: software for RNA secondary structure prediction and analysis. BMC Bioinformatics. 11:129.
29. Tamura (2011) MEGA5: Molecular Evolutionary Genetics Analysis Using Maximum Likelihood, Evolutionary Distance, and Maximum Parsimony Methods. Mol. Biol. Evol. 28(10):2731-2739.
30. Rozen S, Skaletsky H (2000) Primer3 on the WWW for general users and for biologist programmers. Methods Mol Biol 132:365-386
31. Schwarz-Sommer Z, Leclercq L, Göbel E, Saedler H (1987) Cin4, an insert altering the structure of the A1 gene in Zea mays, exhibits properties of nonviral retrotransposons. EMBO J 6:3873
32. Liu HM, Wang XW, Wei B, Wang YB, Liu YH, Zhang JJ, Hu YF, Yu GW, Li J, Xu ZB, Huang YB (2016) Characterization of Genome-Wide Variation in Four-Row Wax, a Waxy Maize Landrace with a Reduced Kernel Row Phenotype. Front Plant Sci. 7: 1-12.
33. Pickersgill B (2007) Domestication of plants in the Americas: insights from Mendelian and molecular genetics. Ann Bot. 100: 925-940.
34. Zhang ZP, Li MX (1990) Comparative studies on karyotypes and Giemsa C banding patterns of 8 subspecies (type) in maize. Acta Genet Sin 17: 86–93. (In Chinese).
35. Huang GQ (2016) Planting food making and sacrifice giving with glutinous rice among ethnic groups of the zhuang-dong linguistic family in China. Ethnic Studies in Guangxi 127:80-87
36. Yang ZH (2013) Sticky Rice: A Perspective to Research Ethnic Minorities’ History and Culture in South China. Ethnic Studies in Guangxi 111:109-117
37. Thomas W, François S, Aurélie HV, Jeffrey LB, Pierre C, Boulos C, Andrew F, Philippe L, Michele M, Olivier P, Etienne P, Phillip S, Alan HS (2007) A unified classification system for eukaryotic transposable elements. Nat Rev Genet 8: 973-982
38. Kumar A, Bennetzen JL (1999) Plant retrotransposons. Annu Rev Genet 33(1): 479-532
39. Feschotte C, Jiang N, Wessler SR (2002) Plant transposable elements: where genetics meets genomics. Nat Rev Genet 3: 329-341
40. Chen JJ, Hu Q, Zhang Y, Lu C, Kuang HH (2013) P-MITE: a database for plant miniature inverted-repeat transposable elements. Nucleic Acids Res 42: D1176-D1181
41. Luan D, Korman M, Jakubczak J, Eickbush T (1993) Reverse transcription of R2Bm RNA is primed by a nick at the chromosomal target site: a mechanism for non-LTR retrotransposition. Cell 72: 595-605
42. Cost G, Feng Q, Jacquier A, Boeke J (2002) Human L1 element target-primed reverse transcription in vitro. EMBO J 21: 5899-5910
43. Cordaux R, Batzer M (2009) The impact of retrotransposons on human genome evolution. Nat Rev Genet 10: 691-703
44. Zingler N, Willhoeft U, Brose H, Schoder V, Jahns T, et al. (2005) Analysis of 5' junctions of human LINE-1 and Alu retrotransposons suggests an alternative model for 5'-end attachment requiring microhomology-mediated end-joining. Genome Res 15: 780
45. Wessler S, Tarpley A, Purugganan M, Spell M, Okagaki R (1990) Filler DNA is associated with spontaneous deletions in maize. Proc Natl Acad Sci USA. 87: 8731-8735
46. Wicker T, Yu YS, Haberer G, F.X. Mayer K, Marri PR, Rounsley S, Chen MS, Zuccolo A, Panaud O, A. Wing R, Roffler S (2016) DNA transposon activity is associated with increased mutation rates in genes of rice and other grasses. Nature Commun 7:12790

**Figure 1. Known *waxy* alleles in waxy maize landraces.**

In maize, the wild-type *waxy* gene is about 4.5 kb and has 14 exons numbered e1 to e14. The arrows above the schematic of the gene point to insertion mutations; the lines below indicate deletion mutations. Allele *wx-Cin4-2* is a 1,267 bp insertional mutation in intron 10. Allele *wx-D11* is a 1,082 bp deletion mutation from exon 11 to exon 14.

**Figure 2. Geographic distribution of experimental materials used in this study.**

(a) Shows the location of Yunnan in China, (b) shows the location of the source of materials in Yunnan. ▲Zinuoyumi came from Lancang County; ●Four-Row Wax came from Menghai, Menglian, Yingjiang and Namhkan areas of the China-Myanmar border.

**Figure 3. Spike morphology of materials used in this study.**

(a) is purple ears of Zinuoyumi; (b) is purple cob of Zinuoyumi; (c) is ear front of Myanmar Four-Row Wax; (d) is ear back of Myanmar Four-Row Wax. Bar = 2 cm.

**Figure 4. Sequence and structure of the *Cin4* in *wx-Cin4-2*.**

The full-length *Cin4* transposon is about 7 kb in length and has two open reading frames (ORF) to encode the necessary enzymes for jumping in maize genome. The length of *Cin4* in *wx-Cin4-2* is 1,267 bp, which is a truncated transposon of *Cin4*. Both full-length and non-full-length *Cin4* have target site duplications (TSDs) sequence and poly (A) tail at 3’ end.

**Figure 5. The NG tree of *Cin4* from *wx-Cin4*, *wx-Cin4-2* and 23 *Cin4* from** **B73 Genome.**

The *Cin4* from *wx-Cin4* and *wx-Cin4-2* is divided on the same branch as the full-length *Cin4* *Cin4-2*.

**Figure 6. The extra sequence of *Cin4*.**

(a) Sequence alignment of *wx-Cin4* and *Cin4-2*; (b) Sequence alignment of *wx-Cin4-2* and *Cin4-2*; (c) the structure of extra sequence. There were overlapping sequences between the extra sequences, the target sequence and *Cin4* sequence. The red box indicated that the 5' end sequences of *wx-Cin4-2* and *wx-Cin4* were found not to match *Cin4-2*, which are extra sequences of *Cin4*.

**Figure 7. The filler sequence structure of *wx-D11*.**

The filled sequence has 11 bp TSDs structure 5’-TGGTACGTGTG -3’. The filler sequence between TSDs can be divided into three parts: Part I is an 18 bp forward sequence from exon 10 of *waxy* gene 5’- CGCCGCCCGCCACCCGGC -3’; Part II is a 17 bp unknown source sequence 5’- CACCACAAGCACACTAT -3’; Part III is a 37 bp reverse complementary sequence from exon 14 of *waxy* gene 5’-TATATTACACTAGCACAAGCAAGCAGCTACACATACT -3’.

**Figure 8. The 1% agarose gel electrophoresis results of the simplified the *wx-Cin4-2* and the *wx-D11*.**

(a), the *wx-Cin4-2* from Zinuoyumi; (b), the *wx-D11* from Myanmar Four-Row Wax.

**Figure 9. The possible process of *Cin4* jump.**

(a) The first strand was cleaved at the transposition target site, producing a free hydroxyl (OH) at the nick. (b) The cDNA synthesis used the free hydroxyl (OH) as a primer following template RNA, and the second strand was cleaved at transposition target site. (c) Template RNA leaved from the cDNA during the synthesis of cDNA. After the 3’ end of cDNA annealed to a new site of the template RNA, cDNA synthesis restarted. (d) The cleavage of the second strand annealed to cDNA, the second strand synthesis started following newly cDNA. (e) A copy of element was integrated at a new genomic location and was flanked by target site duplications (TSDs). The extra sequence is formed in process (c) and remains in *Cin4* after transposition.

**Figure 10. The secondary structure of the *wx-D11* deletion sequence (1082 bp).**

The deletion sequence can constitute a stem-loop structure with arms and rings.

**Table 1. Specific molecular markers for detecting different *waxy* alleles.**

**Table 2. Molecular markers that can cover the entire *waxy* gene used in this study.**

**Table 3. The non-LTR retrotransposon *Cin4* in B73** **genome.**

**Table4. The copy number of transposon found in different *waxy* alleles in the B73 genome	.**

**Supplementary1. 53 sequences of *Cin4* from B73 genome.**

**Supplementary2. Gene mutation site sequence of *wx-Cin4-2* and** ***wx-D11.***

**Supplementary3. Deletion sequence of *wx-D11.***
