# Supplementary material for: Structure characteristics of mutation sites in two waxy alleles from Yunnan waxy maize (Zea mays L. var. certaina Kulesh) landraces
Source: PLoS One. 2023 Sep 8;18(9):e0291116. doi: 10.1371/journal.pone.0291116 (PMC10490952; doi:10.1371/journal.pone.0291116)
Supplement: Attachment — Submitted filename: Response to Reviewers.doc [file pone.0291116.s003.doc]

Journal Requirements:

When submitting your revision, we need you to address these additional requirements.

1. Please ensure that your manuscript meets PLOS ONE's style requirements, including those for file naming. The PLOS ONE style templates can be found at 
https://journals.plos.org/plosone/s/file?id=wjVg/PLOSOne_formatting_sample_main_body.pdf and 
https://journals.plos.org/plosone/s/file?id=ba62/PLOSOne_formatting_sample_title_authors_affiliations.pdf

**Response:**

Thank you for your constructive comments.

We have revised the full text format according to the PLOS ONE style.

2. We noticed you have some minor occurrence of overlapping text with the following previous publication(s), which needs to be addressed:

https://link.springer.com/article/10.1007/s10722-019-00763-z?code=2dc26c94-5697-460e-9d56-b1f22a4601f3&error=cookies_not_supported 
https://repository.cimmyt.org/xmlui/bitstream/handle/10883/3264/97999.pdf?isAllowed=y&sequence=1

In your revision ensure you cite all your sources (including your own works), and quote or rephrase any duplicated text outside the methods section. Further consideration is dependent on these concerns being addressed.

**Response:**

Thank you for your constructive comments.

We have revised the overlapping text including Line#109~112 and Line#174.

3. PLOS ONE now requires that authors provide the original uncropped and unadjusted images underlying all blot or gel results reported in a submission’s figures or Supporting Information files. This policy and the journal’s other requirements for blot/gel reporting and figure preparation are described in detail at https://journals.plos.org/plosone/s/figures#loc-blot-and-gel-reporting-requirements and https://journals.plos.org/plosone/s/figures#loc-preparing-figures-from-image-files. When you submit your revised manuscript, please ensure that your figures adhere fully to these guidelines and provide the original underlying images for all blot or gel data reported in your submission. See the following link for instructions on providing the original image data: https://journals.plos.org/plosone/s/figures#loc-original-images-for-blots-and-gels. 
  
In your cover letter, please note whether your blot/gel image data are in Supporting Information or posted at a public data repository, provide the repository URL if relevant, and provide specific details as to which raw blot/gel images, if any, are not available. Email us at [plosone@plos.org](mailto:plosone@plos.org) if you have any questions.

**Response:**

Thank you for your constructive comments.

We have provided the original uncropped and unadjusted images Fig 8A_raw_images.pdf and Fig 8B_raw_images.pdf

4. Thank you for stating the following financial disclosure:

This work was supported by the National Natural Science Foundation of China (32060459)
  
Please state what role the funders took in the study.  If the funders had no role, please state: "The funders had no role in study design, data collection and analysis, decision to publish, or preparation of the manuscript." 
If this statement is not correct you must amend it as needed. 
Please include this amended Role of Funder statement in your cover letter; we will change the online submission form on your behalf.

**Response:**

Thank you for your constructive comments.

We have stated "The funders had no role in study design, data collection and analysis, decision to publish, or preparation of the manuscript.” on Lin # 427.

5. We note that you have stated that you will provide repository information for your data at acceptance. Should your manuscript be accepted for publication, we will hold it until you provide the relevant accession numbers or DOIs necessary to access your data. If you wish to make changes to your Data Availability statement, please describe these changes in your cover letter and we will update your Data Availability statement to reflect the information you provide.

**Response:**

Thank you for your constructive comments.

The gene sequence in this article has been submitted to the genebank database and the sequence number *wx-Cin4-2* (OR161363) *wx-D11* (OR161364) has been obtained.

6. PLOS requires an ORCID iD for the corresponding author in Editorial Manager on papers submitted after December 6th, 2016. Please ensure that you have an ORCID iD and that it is validated in Editorial Manager. To do this, go to ‘Update my Information’ (in the upper left-hand corner of the main menu), and click on the Fetch/Validate link next to the ORCID field. This will take you to the ORCID site and allow you to create a new iD or authenticate a pre-existing iD in Editorial Manager. Please see the following video for instructions on linking an ORCID iD to your Editorial Manager account: <https://www.youtube.com/watch?v=_xcclfuvtxQ>

**Response:**

Thank you for your constructive comments.

The corresponding author has provided the ORCID iD.

7. We note that Figure 2 in your submission contain map images which may be copyrighted. All PLOS content is published under the Creative Commons Attribution License (CC BY 4.0), which means that the manuscript, images, and Supporting Information files will be freely available online, and any third party is permitted to access, download, copy, distribute, and use these materials in any way, even commercially, with proper attribution. For these reasons, we cannot publish previously copyrighted maps or satellite images created using proprietary data, such as Google software (Google Maps, Street View, and Earth). For more information, see our copyright guidelines: http://journals.plos.org/plosone/s/licenses-and-copyright.
 
We require you to either (1) present written permission from the copyright holder to publish these figures specifically under the CC BY 4.0 license, or (2) remove the figures from your submission:
 
a. You may seek permission from the original copyright holder of Figure 2 to publish the content specifically under the CC BY 4.0 license.  
 
We recommend that you contact the original copyright holder with the Content Permission Form (http://journals.plos.org/plosone/s/file?id=7c09/content-permission-form.pdf) and the following text:
“I request permission for the open-access journal PLOS ONE to publish XXX under the Creative Commons Attribution License (CCAL) CC BY 4.0 (http://creativecommons.org/licenses/by/4.0/). Please be aware that this license allows unrestricted use and distribution, even commercially, by third parties. Please reply and provide explicit written permission to publish XXX under a CC BY license and complete the attached form.”
 
Please upload the completed Content Permission Form or other proof of granted permissions as an "Other" file with your submission.
 
In the figure caption of the copyrighted figure, please include the following text: “Reprinted from [ref] under a CC BY license, with permission from [name of publisher], original copyright [original copyright year].”
 
b. If you are unable to obtain permission from the original copyright holder to publish these figures under the CC BY 4.0 license or if the copyright holder’s requirements are incompatible with the CC BY 4.0 license, please either i) remove the figure or ii) supply a replacement figure that complies with the CC BY 4.0 license. Please check copyright information on all replacement figures and update the figure caption with source information. If applicable, please specify in the figure caption text when a figure is similar but not identical to the original image and is therefore for illustrative purposes only.
The following resources for replacing copyrighted map figures may be helpful:
 
USGS National Map Viewer (public domain): http://viewer.nationalmap.gov/viewer/
The Gateway to Astronaut Photography of Earth (public domain): http://eol.jsc.nasa.gov/sseop/clickmap/
Maps at the CIA (public domain): https://www.cia.gov/library/publications/the-world-factbook/index.html and https://www.cia.gov/library/publications/cia-maps-publications/index.html
NASA Earth Observatory (public domain): http://earthobservatory.nasa.gov/
Landsat: http://landsat.visibleearth.nasa.gov/
USGS EROS (Earth Resources Observatory and Science (EROS) Center) (public domain): http://eros.usgs.gov/#
Natural Earth (public domain): <http://www.naturalearthdata.com/>

**Response:**

Thank you for your constructive comments.

We have chosen to delete the Figure 2

8. Please include your tables as part of your main manuscript and remove the individual files. Please note that supplementary tables (should remain/ be uploaded) as separate "supporting information" files

9. Please review your reference list to ensure that it is complete and correct. If you have cited papers that have been retracted, please include the rationale for doing so in the manuscript text, or remove these references and replace them with relevant current references. Any changes to the reference list should be mentioned in the rebuttal letter that accompanies your revised manuscript. If you need to cite a retracted article, indicate the article’s retracted status in the References list and also include a citation and full reference for the retraction notice.

**Response:**

Thank you for your constructive comments.

We have revised the references carefully.

[Note: HTML markup is below. Please do not edit.]

Reviewers' comments:

Reviewer's Responses to Questions

**Comments to the Author**

1. Is the manuscript technically sound, and do the data support the conclusions?

The manuscript must describe a technically sound piece of scientific research with data that supports the conclusions. Experiments must have been conducted rigorously, with appropriate controls, replication, and sample sizes. The conclusions must be drawn appropriately based on the data presented.

Reviewer #1: Yes

Reviewer #2: Yes

Reviewer #3: Yes

2. Has the statistical analysis been performed appropriately and rigorously?

Reviewer #1: Yes

Reviewer #2: Yes

Reviewer #3: N/A

3. Have the authors made all data underlying the findings in their manuscript fully available?

The [PLOS Data policy](http://www.plosone.org/static/policies.action#sharing) requires authors to make all data underlying the findings described in their manuscript fully available without restriction, with rare exception (please refer to the Data Availability Statement in the manuscript PDF file). The data should be provided as part of the manuscript or its supporting information, or deposited to a public repository. For example, in addition to summary statistics, the data points behind means, medians and variance measures should be available. If there are restrictions on publicly sharing data—e.g. participant privacy or use of data from a third party—those must be specified.

Reviewer #1: Yes

Reviewer #2: Yes

Reviewer #3: Yes

4. Is the manuscript presented in an intelligible fashion and written in standard English?

PLOS ONE does not copyedit accepted manuscripts, so the language in submitted articles must be clear, correct, and unambiguous. Any typographical or grammatical errors should be corrected at revision, so please note any specific errors here.

Reviewer #1: Yes

Reviewer #2: Yes

Reviewer #3: No

5. Review Comments to the Author

Please use the space provided to explain your answers to the questions above. You may also include additional comments for the author, including concerns about dual publication, research ethics, or publication ethics. (Please upload your review as an attachment if it exceeds 20,000 characters)

Reviewer #1: Dear Prof. Dr. Editor of the PLOS ONE Journal,

I write you regarding Manuscript ID: PONE-D-23-09518 entitled "Structure Characteristics of Mutation Sites in Two Waxy Alleles from Yunnan Waxy Landraces" which was submitted to the PLOS ONE Journal.

In this manuscript, the authors studied the Structure Characteristics of Mutation Sites in Two Waxy Alleles from Yunnan Waxy Landraces.

This work was well done and appropriate. The manuscript is suitable for publication in the International Journal of Molecular Sciences.

I have gone through this work. My decision is accepted with minor revisions for this work. The reason for that is as follows:
The manuscript deals with " Structure Characteristics of Mutation Sites in Two Waxy Alleles from Yunnan Waxy Landraces".

First: Title: It should change to the following:
1) Structure Characteristics of Mutation Sites in Two Waxy Alleles of Waxy Maize (Zea mays L.) Landraces from Yunnan
**Response:**

Thank you for your constructive comments.

We have revised the title of this manuscript.

Second Abstract, keywords and Introduction:
2) has some minor corrections as in the attached file.
**Response:**

Thank you for your constructive comments.

Third: The other parts
3) has some minor corrections as in the attached file.
**Response:**

Thank you for your constructive comments.

References
4) has some minor corrections as in the attached file.
**Response:**

Thank you for your constructive comments.

Thank you for suggesting me as a reviewer for this manuscript.

with best regards

Reviewer #2: Paper entitled" Structure Characteristics of Mutation Sites in Two Waxy Alleles from Yunnan Waxy
Landraces" was in the scope of journal and writien in scientific language with good English editing. Study the waxy genes is very important for Maize production and increasing the yield productivity. I recommend publication of this manuscript after minor revision.
Introduction. Need more explanation in the role of waxy genes in productivity of Maize and other phenotypic characters.

**Response:**

Thank you for your constructive comments.

We added the role of the waxy gene in the starch synthesis pathway in the introduction.

Results. You can characterize the Waxy genes in genbank database and compare it with the genes present in the data base

**Response:**

Thank you for your constructive comments.

We compared the characteristics of different types of waxy alleles in Table 4.

Discussion. Needs more explanation for the genes and compare it with previous study

**Response:**

Thank you for your constructive comments.

The “Transposon activity is the main driving force for the formation of waxy allele diversity” part in discussed of this article was compared with our previous work.

Reviewer #3: The manuscript entitled “Structure Characteristics of Mutation Sites in Two Waxy Alleles from Yunnan Waxy Landraces", is well-written with an extensive discussion and addresses the main objectives of the study. It provides valuable insights into the Structural Characteristics of Waxy genes. In my review, I carefully and thoroughly assessed the content of the report, also, I have identified several key points that I believe are important to highlight. However, there are areas where improvements can be made, that may benefit from further clarification or revision of the article (Manuscript ID: PONE-D-23-09518- PLOS ONE). The suggestions below are:

Line#93 (Similarly, for line#132), Author stated that “two different mutation sites and 30bp deletion in exon 10” in wx-Cin4-2 of Zinuoyumi. However, it not clearly mentioned or work done anywhere in the entire manuscript, with reference to what, author stated two mutation sites and deletion30bp? What kind of mutation is (sense or nonsense, homo or heterozygous)? What is the consequence of these mutations, although author has partly explained in the discussion section, but need to explain in detail. Because "sense" or "nonsense" describes the impact of a mutation on protein function, while "homozygous" or "heterozygous" refers to the genetic state of an individual with respect to the mutation. These terms are distinct and address different aspects of genetic mutations.
Additionally, as author has shown specificity on agarose gel, I don’t see sanger sequencing confirmed mutation using chromatogram. That will help to assess mutation type and other.

**Response:**

Thank you for your constructive comments.

The 30bp deletion in exon 10 is a type of mutation found in previous studies. Besides the 30bp deletion in exon 10, *wx-Cin4-2* also has the transposable element *Cin4* insertion mutation.

Line#119, Author stated, “The wx-Cin4-2 has been identified only in Zinuoyumi. Two waxy alleles caused by Cin4 transposon insertion were identified in Zinuoyumi, suggesting that maybe active Cin4 transposons exist in genome of Zinuoyumi. However, what is the advantage of having this mutation in Zinuoyumi is not clear, whether it has any survival advantage association.

**Response:**

Thank you for your constructive comments.

There are still active Transposable element in the genome of Landrace of waxy maize in Yunnan, perhaps Cin4 in the genome of Zinuoyumi is one of them.

Line#146-147
Yunnan and its surrounding areas are the secondary origin center of waxy maize Vavilov’s theory on origin center of crops: Why half is bold, author need to continue and reframe sentence without colon sign”:” and bold.

Response:

Thank you for your constructive comments.

We have modified the font.
Line#146-159, the reference is missing in the entire section.

**Response:**

Thank you for your constructive comments.

We have added references.
The entire discussion in the manuscript is so lengthy and there is lot of irrelevant matter and can be rearranged from discussion part to the introduction section for example Line #178-193 can be added to the introduction part but just brief little in discussion rather lengthy.

Line#44, why author has started sentence with “AND” as it can be use and to connect two words, phrases, clauses or prefixes together, Author needs to correct this.
**Response:**

Thank you for your constructive comments.

We have modified this sentence.
Line #46 and 48, Author needs to reframe the sentence “ The known alleles include wx-D7, wx-D10, wx-Cin4, wx-124, wx-Reina, wx-Xuanwei, wx PIF/Harbinger, wx-hAT, and wx-Elote2 [13-16]; wx-D7 and wx-D10 “were mutations” caused by sequence deletion in waxy gene [17, 18]; wx-Cin4, wx-124, wx-Reina, wx-Xuanwei, wx-PIF/Harbinger, wx-hAT, and wx-Elote2 “are mutations” caused by transposon insertion in waxy gene [13-16]; wx-D10 and wx-Reina are widely distributed waxy alleles in waxy maize landraces of Yunnan area.
**Response:**

Thank you for your constructive comments.

We have modified this sentence.
Instead, author should write “mutations were” caused by sequence deletion in waxy gene [17, 18], and similarly, , wx-hAT, and wx-Elote2 “having mutations.”
**Response:**

Thank you for your constructive comments.

We have modified this sentence.
Similarly, author stated that, previous studies have found that wx-B, wx-BI, wx-B6, and wx-C4 “are” mutations due to waxy gene sequence deletions from exons 1 to 7, and the deletion sequences are replaced by filling sequences. Author should reframe “are” instead use “have”.
**Response:**

Thank you for your constructive comments.

We have modified this sentence.
Line# 54-55, Author has stated that “The identification of waxy allele is helpful to the protection and utilization of these waxy landraces” However, it seems incomplete sentence, what author want to state here, not clear. Similarly, Author stated that, “large number of Yunnan waxy maize landraces still contain unknown waxy alleles that need to be identified, I am wondering how author knows about this is not clear, whether it is functional characterization terms or some other mean and promoter has not been assigned in figure 1 although CCAAT and TATAA box has being mentioned and The major thing I don’t see is the functional association with these mutations is missing.

**Response:**

Thank you for your constructive comments.

We have modified this sentence.

We have modified this sentence.

The CCAAT and TATAA box represent the location of gene transcription initiation.

6. PLOS authors have the option to publish the peer review history of their article ([what does this mean?](https://journals.plos.org/plosone/s/editorial-and-peer-review-process#loc-peer-review-history)). If published, this will include your full peer review and any attached files.


If you choose “no”, your identity will remain anonymous but your review may still be made public.


**Do you want your identity to be public for this peer review?** For information about this choice, including consent withdrawal, please see our [Privacy Policy](https://www.plos.org/privacy-policy).

Reviewer #1: **Yes:** Khaled F. M. Salem

Reviewer #2: No

Reviewer #3: **Yes:** MUDASIR RASHID

[NOTE: If reviewer comments were submitted as an attachment file, they will be attached to this email and accessible via the submission site. Please log into your account, locate the manuscript record, and check for the action link "View Attachments". If this link does not appear, there are no attachment files.]

While revising your submission, please upload your figure files to the Preflight Analysis and Conversion Engine (PACE) digital diagnostic tool, <https://pacev2.apexcovantage.com/>. PACE helps ensure that figures meet PLOS requirements. To use PACE, you must first register as a user. Registration is free. Then, login and navigate to the UPLOAD tab, where you will find detailed instructions on how to use the tool. If you encounter any issues or have any questions when using PACE, please email PLOS at [figures@plos.org](mailto:figures@plos.org). Please note that Supporting Information files do not need this step.
